# Opening up the blackbox: an interpretable deep neural network-based classifier for cell-type specific enhancer predictions

**DOI:** 10.1186/s12918-016-0302-3

**Published:** 2016-08-01

**Authors:** Seong Gon Kim, Nawanol Theera-Ampornpunt, Chih-Hao Fang, Mrudul Harwani, Ananth Grama, Somali Chaterji

**Affiliations:** Department of Computer Science, Purdue University, West Lafayette, IN USA

**Keywords:** Genomic enhancers, Enhancer prediction, Deep neural networks (DNNs), Histone modifications, ChIP-seq, Cis-regulatory modules (CRMs), Interpretability of blackbox models

## Abstract

**Background:**

Gene expression is mediated by specialized cis-regulatory modules (CRMs), the most prominent of which are called *enhancers*. Early experiments indicated that enhancers located far from the gene promoters are often responsible for mediating gene transcription. Knowing their properties, regulatory activity, and genomic targets is crucial to the functional understanding of cellular events, ranging from cellular homeostasis to differentiation. Recent genome-wide investigation of epigenomic marks has indicated that enhancer elements could be enriched for certain epigenomic marks, such as, combinatorial patterns of histone modifications.

**Methods:**

Our efforts in this paper are motivated by these recent advances in epigenomic profiling methods, which have uncovered enhancer-associated chromatin features in different cell types and organisms. Specifically, in this paper, we use recent state-of-the-art Deep Learning methods and develop a deep neural network (DNN)-based architecture, called EP-DNN, to predict the presence and types of enhancers in the human genome. It uses as features, the expression levels of the histone modifications at the peaks of the functional sites as well as in its adjacent regions. We apply EP-DNN to four different cell types: H1, IMR90, HepG2, and HeLa S3. We train EP-DNN using p300 binding sites as enhancers, and TSS and random non-DHS sites as non-enhancers. We perform EP-DNN predictions to quantify the validation rate for different levels of confidence in the predictions and also perform comparisons against two state-of-the-art computational models for enhancer predictions, DEEP-ENCODE and RFECS.

**Results:**

We find that EP-DNN has superior accuracy and takes less time to make predictions. Next, we develop methods to make EP-DNN interpretable by computing the importance of each input feature in the classification task. This analysis indicates that the important histone modifications were distinct for different cell types, with some overlaps, e.g., H3K27ac was important in cell type H1 but less so in HeLa S3, while H3K4me1 was relatively important in all four cell types. We finally use the feature importance analysis to reduce the number of input features needed to train the DNN, thus reducing training time, which is often the computational bottleneck in the use of a DNN.

**Conclusions:**

In this paper, we developed EP-DNN, which has high accuracy of prediction, with validation rates above 90 % for the operational region of enhancer prediction for all four cell lines that we studied, outperforming DEEP-ENCODE and RFECS. Then, we developed a method to analyze a trained DNN and determine which histone modifications are important, and within that, which features proximal or distal to the enhancer site, are important.

## Background

Distinct cell phenotypes are largely modulated by unique gene expression patterns, stemming from the interaction of the genome with its environment. Such crosstalk is mediated by specialized cis-regulatory modules (CRMs), including enhancers [1], silencers, promoters, and insulators [2–4]. Among these, enhancers constitute the most prominent class of gene expression regulators. Early experiments indicated that sequences located far from the gene promoters are often responsible for mediating gene transcription [5]. Such genetic elements are called *enhancers*, defined as short DNA sequences regulating temporal and cell-type specific basal gene-transcription levels, from transcription start sites (TSSs), at distances ranging from hundreds of bases to, in rare cases, even megabases [6–8]. Knowing their properties, regulatory activity, and genomic targets is crucial to the functional understanding of cellular events, ranging from cellular homeostasis to differentiation. Recent genome-wide investigation of epigenomic marks has indicated that enhancer elements could be enriched for certain epigenomic marks, such as complex, albeit predictive, combinatorial signatures of histone modifications. Our efforts in this paper are motivated by these recent advances in epigenomic profiling methods, which have uncovered enhancer-associated chromatin features in different cell types and organisms [9–12]. Specifically, in this paper, we use recent state-of-the-art Deep Learning methods and develop a deep neural network (DNN)-based architecture [13–15] to predict the presence and types of enhancers in the human genome. We call our system “*EP-DNN*”, an acronym for “*Enhancer Prediction Deep Neural Network*”.

Historically, computational identification of enhancers has proven to be challenging for several reasons [16]. First, the search space for enhancers is large—billions of DNA base pairs—scattered across 98 % of the non-coding genome. Second, while enhancers regulate genes in *cis*, they do not display distinct locational or orientation-centric signals relative to the genes that they regulate—potentially located upstream, downstream, or even in introns of the genes that they modulate, often regulating multiple genes [17]. Enhancers function at a distance from their target genes via chromatin loops that bring the enhancers and target genes into proximity [18, 19], or via direct eRNA transcription from the enhancer DNA sequences [20]. Third, although a few computational attempts have been made to elucidate sequence-based signatures of enhancers [21–23], they are very recent, and yet to be widely adopted possibly because of the challenge of building models sophisticated enough to perform the classification task. We empirically validate this by considering some intuitive approaches for discriminating different forms of enhancers and non-enhancers (such as, using statistical distributions of expressions of various histone modifications around the sites of the functional elements) and observe that these will be highly inaccurate.

Several high-throughput *experimental* approaches exist to identify enhancers [23, 24]. The first is mapping specific transcription factor binding sites (TFBS) through ChIP-seq [25]. This stems from the fact that short enhancer DNA sequences serve as binding sites for TFs, and the combined regulatory cues of all bound TFs determine ultimate enhancer activity [26, 27]. However, this approach requires the knowledge of the TF subset that is not only expressed but also occupies all active enhancer regions in the spatiotemporal setting, such as in a specific cell type at a point of time [28]. Therefore, predicting enhancer activity from sequence-based information, such as from the TF motif content, remains challenging [27, 29]. In addition, this approach is limited by the lack of available ChIP-grade antibodies that specifically recognize these subsets. The second approach is based on mapping transcriptional co-activator binding sites (e.g., histone acetyltransferase HAT, *p300*) [30, 31]. However, not all enhancers are marked by a set of co-activators and also often lack available ChIP-grade antibodies. The third approach relies on identifying open chromatin regions by DNase-I hypersensitivity (DHS) mapping [32], which lacks specificity due to the fact that the identified regions can correspond to other CRMs. Finally, the fourth approach involves histone modification signatures produced by ChIP-seq that consistently mark enhancer regions [33–37]. Due to their consistency in marking enhancers, we use histone modification features for our computational prediction of enhancer signatures.

**Fig. 1:**
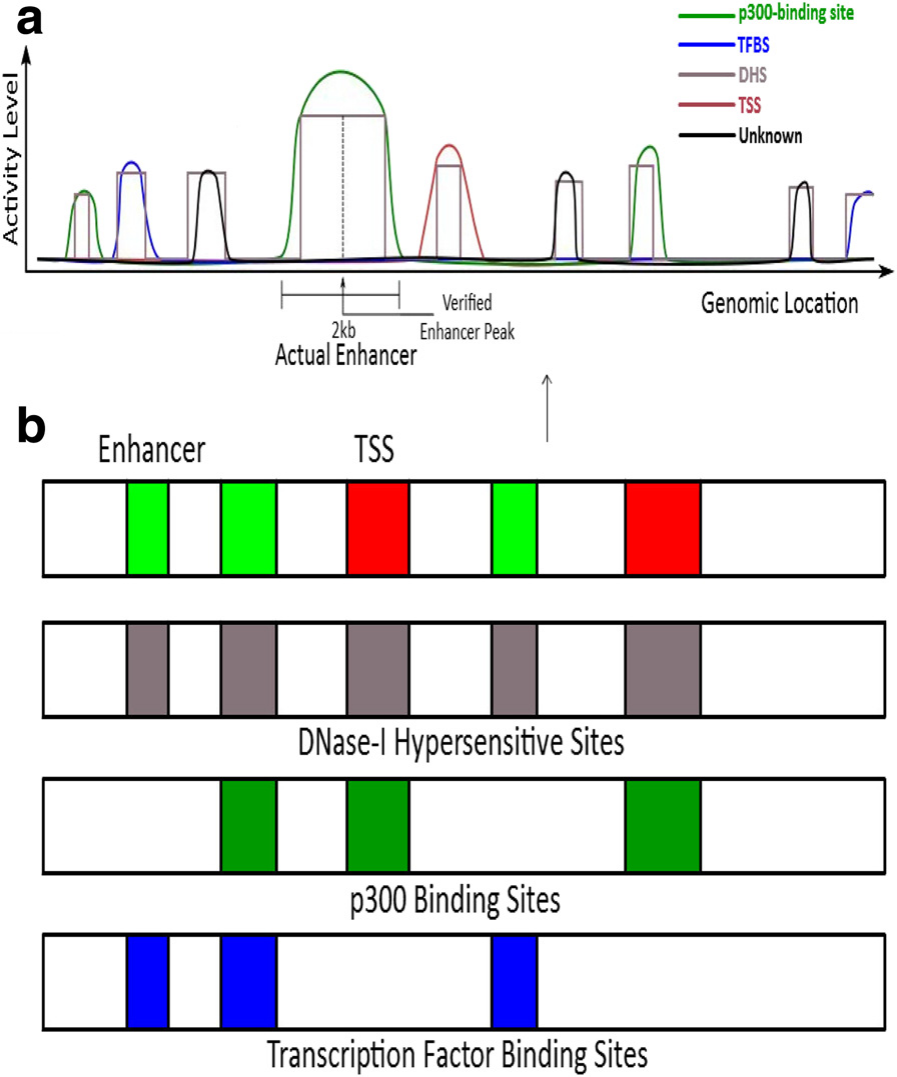
**a** A notional schematic showing the enhancer and the TSS (the promoter) relative to some of the True Positive Markers (TPMs)—DNase-I hypersensitivity site (DHS), p300 binding site, and transcription factor binding site (TFBS) (applicable to the H1 cell line). **b** Various forms of these TPMs overlap with the enhancer and the promoter sites. An overlap of the DHS with the TFBS can indicate an enhancer, while an enhancer is typically distal to the TSS. TPMs refer to DHS, p300, CBP, and TFBS

**Fig. 2:**
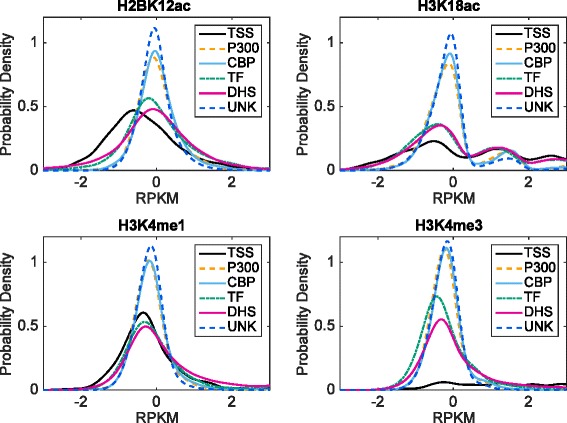
Distributions of ChIP-seq values for each functional type. UNK refers to unknowns. P300, CBP, TF, and DHS are considered positive, while TSS and UNK are considered negative

Through analysis of the prior computational approaches to enhancer prediction, we conclude that the difficulty of computationally predicting enhancer sites is because of two primary factors. First, they did not use the full spectrum of available features, i.e., all the histone modifications and their enrichment values in a wide region around the hypothesized enhancer site, denoted by the enhancer peak. Second, they did not use a highly expressive classifier, one that can extract the distinguishing features from a complex landscape. This complex epigenomic landscape is captured by our empirically-derived distribution of normalized read counts of the four most distinguishing histone modifications for different types of functional sites from embryonic stem cells (H1) [Fig. 1]. The most distinguishing histone modifications are chosen by analyzing the internal weights of trained DNN models. Our empirical plots show that even for the top histone modifications, the overlap between any positive type and unknown is significant, which illustrates that simple rule-based classifiers would not perform well [Fig. 2].

We address both of these problems, the first by starting with an (almost) exhaustive set of features and then doing feature selection through an innovative mechanism, to identify a top-*k* most relevant features. Empirically, the full set has 480 features (for each of H1 and IMR90), 220 features for HepG2, and 180 features for HeLa, and the reduced feature set for each cell line. For the second shortcoming, we use recent state-of-the-art Deep Learning methods and develop a DNN-based architecture [13–15] to predict the presence and types of enhancers in the human genome, “learning” from the combinatorial histone modification codes. We get the histone modification data from NIH Epigenome Roadmap for H1 and IMR90 and from the NHGRI ENCODE database for HepG2 and HeLa S3. The enhancement level is available from ChIP-seq experiments and for our prediction, we bin them into 100 bp windows around the peaks of the regulatory elements, the total extent around the peak being 2 kbp.

**Fig. 3:**
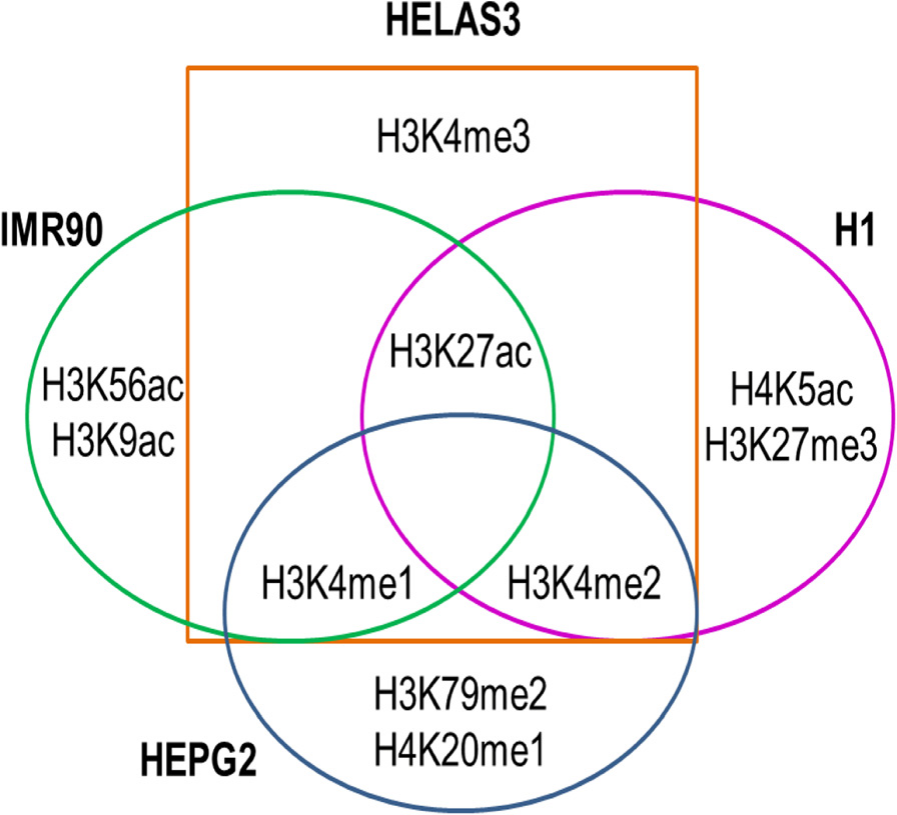
Venn diagram showing overlaps of top-4 histone modification among the four cell types

Next, we adapt a previously proposed theoretical mechanism [38] for interpreting the results of the DNN to rank order the features according to how important they are for performing the classification task. We find that there are certain overlaps between which histone modifications are important for which cells, and even at a finer granularity, which spatial feature is important within each histone modification. For example, if one considers the top-*4* most important histone modifications for each cell type, there is none that is common among the 4 cell types. However, between pairs of cell lines there are some commonalities—H3K18ac is important for both H1 and IMR90, while H3K9ac and H3K79me2 are found to be important for both HepG2 and HeLa S3 [Fig. 3]. We use this weight analysis to address the problem that training a DNN is computationally expensive. We rank order the features by importance and train DNNs using only the top *k* features, for varying values of *k*. We compare the result of the prediction by the reduced DNNs to that of the prediction by the full DNN (the one which employs the full set of features). We find that the accuracy is within 90 % using the reduced *k*-value for each of the 4 cell types.

Summarizing, we make the following contributions in this paper:

1. We bring to bear a powerful data-analytic technique to enhancer prediction, namely, a Deep Neural Network (DNN)-based technique. We train DNN models that outperform prior computational approaches in terms of prediction accuracy for 2 different cell types, H1, an embryonic cell type, and IMR90, a mature cell type.
2. We then make our DNN models interpretable, and by interpreting them, we find cell type-specific differences among our 4 distinct cell types. These differences highlight that different histone modifications are important for the prediction problem in these different cell types and even within a histone modification, which spatial features are important tend to differ.
3. We come up with a method to reduce the computational cost of training our DNN by reducing the input feature space in a data-driven manner.

### Biological use case of our solution, EP-DNN

We hypothesize some possible use cases for EP-DNN. First, an experimentalist by knowing the features and the histone modifications that are important to the task of classification can choose to collect data only for the most important ones among them. Second, our data analytic technique reduces the noise inherent in the biological data by focusing on the important features. With respect to the spatial features, EP-DNN provides an insight into how far away from the peak locations, one needs to consider the effect of a functional element. In a prior approach, a 2 kbp region was used but this was *ad hoc*. Armed with our analysis, any computational procedure, not just ours, can make an informed decision, e.g., only a narrow region (say 500 bp) may be enough for a certain histone modification and a certain type of enhancer, while a wider region may be needed for a different type. Third, we have started the process of identifying overlaps among the different cell types with respect to the important histone modifications. Taking this process further, we can cluster different cell types with respect to their enhancer characteristics by using EP-DNN and then perform extrapolations for data from newer methods, such as, global run-on and sequencing (GRO-seq), chromosome conformation capture (3C) technologies, and their genome-wide derivatives that are used to analyze the activity levels and the 3D structures of the genome, respectively.

### Related work: Previous computational methods based on histone modifications

Won et al. proposed the usage of Hidden Markov Models (HMMs) to predict enhancers using three primary histone modifications [34]. Firpi et al. focused on the importance of recognizing the histone modification signals through data transformation and employed Time-Delayed Neural Networks (TDNNs) using a set of histone marks selected through simulated annealing [35]. Fernández et al. used Support Vector Machines (SVMs) on a subset of histone modifications. The subset had been determined through using Genetic Algorithms [36]. RFECS (Random Forest based Enhancer identification from Chromatin States) improved upon the limited number of training samples in previous approaches using Random Forests (RFs), in order to determine the optimal subset of histone modification signatures in order to predict enhancers [37]. DEEP uses features derived from histone modification marks or attributes coming from sequence characteristics and inputs them in an ensemble prediction framework, which comprises multiple SVMs and an Artificial Neural Network to vote on the results from the SVMs [39]. They show impressive results on three separate databases, ENCODE (DEEP-ENCODE), FANTOM5, and VISTA.

## Methods

**Fig. 4:**
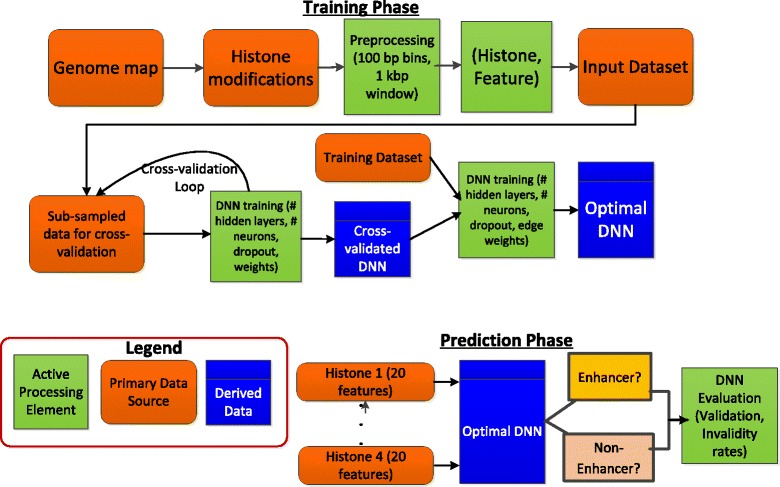
Overview of our solution approach in which we train DNNs using the histone modifications and their associated features. We perform weight analysis and feature selection to identify the optimal DNN, which is then used for predicting if a regulatory region is an enhancer or not

We present a high level overview of our approach in Fig. 4. In the figure, we show, separately, the training phase and the prediction phase. In the training phase, we create an optimal DNN using a set of histone modifications and the associated spatial features, and further, we do the weight analysis to rank order the features by their importance in discriminating the positive and the negative samples. In the prediction phase, we use the same set of features to predict if a regulatory region is an enhancer or not, followed by validation of our results. We predict enhancers in four distinct human cell types—embryonic stem cells (H1), IMR90, HepG2, and HeLa S3, which were generated as a part of the NIH Epigenome Roadmap Project [12] and the NHGRI ENCODE Project [9] [Table 1].

**Table 1:** Description of the 4 different cell lines in our study and the corresponding feature set

Cell type (human)	Number of histone modifications	Number of features	Source of the data
H1 (embryonic stem cell line)	24	24 × 20 = 480	NIH Epigenome Roadmap
IMR90 (fetal lung fibroblast cell line)	24	24 × 20 = 480	NIH Epigenome Roadmap
HepG2 (mature liver cell line)	11	11 × 20 = 220	ENCODE
HeLa (cervical cancer cell line)	9	9 × 20 = 180	ENCODE

### Datasets

The development of EP-DNN is motivated by the availability of data from large scale projects, such as the ENCODE project [9], which has annotated 400,000 putative human enhancers, with the current estimate of enhancer numbers being over a million [40]. Another extensive database is the NIH Roadmap Epigenomics Project [10, 12] that also provides publicly-available epigenomics maps, complementary to ENCODE. In addition, the NCBI’s Gene Expression Omnibus (GEO) repository [11] also hosts much previous work and data on enhancer prediction. We have used data from all 3 of these large-scale data repositories for arriving at our training and validation data for EP-DNN. In particular, we used NIH Roadmap Epigenomics for ChIP-Seq histone modification data for H1 and IMR90, which includes following 24 modifications in BED format: H3K36me3, H3K27me3, H3K4me1, H3K4me3, H3K9ac, H3K9me3, H3K27ac, H2AK5ac, H2AZ, H2BK120ac, H2BK12ac, H2BK15ac, H2BK20ac, H3K18ac, H3K23ac, H3K4ac, H3K4me2, H3K56ac, H3K79me1, H3K79me2, H4K20me1, H4K5ac, H4K8ac, H4K91ac, and DNase I Hypersensitivity Sites (DHS) and Transcription Factor Binding Sites (TFBS) data. The ENCODE ChIP-Seq experiment includes following 11 modifications for HepG2: H2AZ, H3K27me3, H3K4me1, H3K4me3, H3K9me3, H4K20me1, H3K27ac, H3K36me3 H3K4me2, H3K9ac, and H3k79me2 and following 9 for HeLa S3: H3K36me3, H3K4me3, H4K20me1, H3K27ac, H3K4me1, H3K79me2, H3K27me3, H3K4me2, and H3K9ac in BAM or BED format as well as DHS and TFBS data. BAM files were converted to BED files using BEDTools [41]. The p300 binding data for H1 and IMR90 was downloaded from GEO repository GSE37858, generated by Bing Ren’s laboratory. For the TSS locations for HeLa S3 and HepG2, the FANTOM 5 consortium [42] was used, which hosts cell-specific Cap Analysis of Gene Expression (CAGE) data; CAGE measures TSS expression levels by sequencing large amounts of transcript 5′ ends, termed CAGE tags. MACS2 was used to call CAGE peaks, from which we selected the ones overlapping with true TSS CAGE markers, available in the FANTOM database. For p300 data for HeLa S3 and HepG2, we used ENCODE ChIP-Seq data, available from GSE31477.

Transcriptional co-activators—p300 and related acetyltransferases—bind to transcription factor (TF) activation domains and have been found to localize to many active enhancers, but not all [33]. Further, p300 co-activators are ubiquitous, present in all cell types, and control the expression of numerous genes. Therefore, by using p300 enhancer signatures for training, we can also find other types of enhancers (e.g., CBP- or TF-based), generalizing well toward prediction of multiple classes of enhancers. The number of peak calls of functional elements in the dataset used for cell types, H1, IMR90, HeLa S3, and HepG2, is presented in Table 2.

**Table 2:** The number of peak calls of functional elements in the dataset used for training and prediction for the cell types: H1, IMR90, HeLa S3, and Hep G2, obtained through ChIP-seq and DNase-seq

	H1 (100 bp)	IMR90 (100 bp)	HeLa S3 (100 bp)	Hep G2 (100 bp)
DHS	150,729	149,787	N/A	N/A
TSS	9299	8000	21,165	50,070
p300	13,523	52,988	30,004	36,527
CBP	12,958	N/A	N/A	N/A
TF	71,173	N/A	N/A	N/A

### Preprocessing of histone modification inputs

Previous studies indicate H3K4me1, H3K4me2, H3K4me3, and H3K27ac as the top histone modifications [37], indicative as markers of active enhancers. Therefore, we selected them for our EP-DNN model. However, distinct from prior work, we wanted to see if there are other histone modifications that may also be important in the discrimination among enhancers and non-enhancers. Thus, we selected all the remaining 20 histone modifications that are available in the NIH Epigenome Roadmap for H1 and IMR90.

These other histone modifications are: H2AK5ac, H2BK120ac, H2BK12ac, H2BK15ac, H2BK20ac, H2BK5ac, H3K14ac, H3K18ac, H3K23ac, H3K27me3, H3K36me3, H3K4ac, H3K56ac, H3K79me1, H3K79me2, H3K9ac, H3K9me3, H4K20me1, H4K5ac, H4K91ac for H1, and H2AK5ac, H2BK120ac, H2BK12ac, H2BK15ac, H2BK20ac, H3K14ac, H3K18ac, H3K23ac, H3K27me3, H3K36me3, H3K4ac, H3K56ac, H3K79me1, H3K79me2, H3K9ac, H3K9me3, H4K20me1, H4K5ac, H4K8ac, H4K91ac for IMR90. For the HepG2 cell type, we only had access to a smaller number of histone modifications, 11 in all. These are: H2AZ, H3K27ac, H3K27me3, H3K36me3, H3K4me1, H3K4me2, H3K4me3, H3K79me2, H3K9ac, H3K9me3, and H4K20me1. Also, for the HeLa cell type, we only had access to 9 histone modifications. These are: H3K27ac, H3K27me3, H3K36me3, H3K4me1, H3K4me2, H3K4me3, H3K79me2, H3K9ac, and H4K20me1.

The ChIP-seq reads of these histone modifications were binned into 100 bp intervals and normalized against its corresponding inputs by using an RPKM (reads per kilobase per million) measure. Multiple replicates of histone modifications were used to minimize batch-related differences, and the RPKM-levels of the replicates were averaged to produce a single RPKM measurement per histone modification. We will refer to this enrichment level of a histone modification as its signature. The histone modification signatures of each bin location are then used as input to the DNN.

A notional schematic of the enhancer and the TSS (promoter) relative to the various relevant sites—DHS, TFBS, and p300 is given in Fig. 1. The bounding box is the DHS and we are only considering sites that are overlapping with the DHS. The peak location is shown for each element and the activity level curve is shown on both sides of the peak region.

### Deep Neural Network (DNN) model

DNNs have the traditional advantage that they provide feature extraction capabilities and do not require manual feature engineering or transformation of the data, which in turn would have required domain knowledge. EP-DNN was found to be less computationally expensive than the larger ensemble methods that combine multiple algorithms (e.g., DEEP-ENCODE, which uses multiple SVMs with an ANN decision mechanism [43]) or multiple models of the same kind (e.g., Random Forest in RFECS, which uses multiple decision trees [37]). We experimentally show this higher computational cost of DEEP-ENCODE (DEEP-EN) and RFECS in our Results Section, when performing detailed evaluation of our method.

To train our DNN, we first select distal p300 co-activator binding sites through ChIP-seq, then further select though overlapping DHSs that are distal to TSS, as regions representing enhancers [Fig. 1]. Of these, 5,899 p300 peak calls were selected for H1 and 6,000 peaks for the IMR90 cell line to represent enhancers for the training set. However, p300 co-activators also bind to distal TSSs, which are not enhancers. Therefore, we also select TSS that overlap with DHS, as well as random 100 bp bins that are distal to known DHS or TSS to represent non-enhancers. We include 9,299 TSS peaks from H1 and 8,000 peaks from IMR90 in our training set to distinguish between p300 binding sites that are enhancers and TSS that are not, and 31,994 random distal background sites were selected for H1 and 34,000 for IMR90 to represent non-enhancers for training.

For testing the DNN, we used all known distal p300 and CBP co-activator and TFBS that overlap with DHS as positive enhancer sites, and TSS as non-enhancer sites.

A fully connected DNN with 480 inputs, 1 output, and *softplus* activation functions for each neuron was used to make enhancer predictions. Each input sample consists of *K* number of 20-dimensional vectors of 100 bp bin RPKM-levels, windowed from −1 to +1 kb at each bin location. Each 20-dimensional vector corresponds to a histone modification. This gives a total of 480 features in all for the full DNN for H1 and IMR90, with 24 histone modifications used for those. Training was done in mini batches of 100 samples via stochastic gradient descent. To prevent overfitting, *dropout* training [44] was applied, with a dropout rate of 0.5. An optimal architecture of 3 hidden layers, comprising of 600 neurons in the first layer, 500 in the second, and 400 in the third, was found through cross-validation on half the training data, selected randomly. The full training set was used to train the model before prediction. A convergence on the mean squared error could be achieved with only 5 epochs of training. This extensive training mechanism was found to be suitable to optimize the DNN with its fairly large parameter space.

### Training and prediction

For training, we found that best results were obtained when the ratio of the number of positive and negative training examples was 1:10. The class distribution was not modified during testing. Prediction accuracy is evaluated using 5-fold cross-validation. We compare results for within-cell type prediction, i.e., we train on cell type C1 and predict on the same cell type C1.

*Evaluation Metrics*: The standard precision and recall metrics misrepresent actual prediction performance on real data, since there are many more unknown functional sites than just the p300, CBP, NANOG, SOX2, OCT4 binding enhancers or TSS. Ideally, we would have to evaluate performance on all these sites that are unaccounted for. However, most are not experimentally verified and are unknown. Thus, there is not enough data to make an accurate evaluation of the precision and recall of any computational model. This observation has been made by prior computational approaches for enhancer prediction, such as RFECS. Consequently, they have also not used the standard precision and recall metrics in their evaluation. Furthermore, functional enhancers are experimentally verified by single peak locations. However, in reality, enhancers exist in various levels (heights) and sizes (widths) that more or less gradually decrease around the peaks. These peaks are not available during prediction on real data because we are trying to predict for locations that have not yet been experimentally verified. Therefore, any computational model must be able to predict for the peak as well as the surrounding non-peak regions. Further, the evaluation method must synthesize some criterion to determine what is the ground truth (is it an enhancer or not) for any genic region away from the peak location. Therefore, the traditional evaluation using precision and recall metrics cannot be used in this case.

However, once a positive enhancer prediction has been made, it can be *validated*, and the metric that we use to compare the performance of EP-DNN is the *validation rate*. This metric has been used previously for evaluation of enhancer prediction, in RFECS [37], and we modify it slightly here. In our definition, we refer to True Positive markers (TPM) as distal DHS sites, p300, CBP, and TFBS that are greater than 1 kb away from TSS.

- If a predicted enhancer lies within 2.5 kb of a TPM, then EP-DNN’s prediction is “validated”. In this case, we know that this site is either a known or an unknown enhancer, safely assumed to be an enhancer since it overlaps with a DHS site.
- Otherwise, EP-DNN’s prediction is “invalidated”. This means that it is either a TSS or an Unknown, but we know for a fact it is not an enhancer.

The modification from RFECS’ validation is that they had a separate class called unknown and we do not. We categorize it as a mistake if an unknown category is predicted by us as an enhancer. This is because while its exact functional characteristic is unknown, what is known for certainty is that it is *not* an enhancer. Hence, predicting this as an enhancer is an error.

As DNN’s output is a number (whose range varies depending on the activation function that is used), we need to convert it to a class label by comparing it with a threshold. By varying the threshold, we can control the tradeoff between the number of enhancers being predicted and the validation rate. In general, as the threshold is increased, the number of enhancers being predicted goes down and the validation rate goes up. To compare against previous algorithms, in the first experiment, we used the same training and testing datasets for H1 and IMR90 with RFECS and DEEP-EN, the competitive approaches, as for EP-DNN. However, for HeLa S3 and HepG2, we used a smaller dataset in the interest of computational time (Table 1).

### Reduced DNN

Since the cost of training an entire DNN with the total set of inputs—480 features for each of H1 and IMR90, 220 for HepG2 and 180 for HeLa—can be significant, we take a principled approach to reducing the number of input features without significantly affecting the accuracy of our prediction. We refer to the DNN with the complete set of 480 features as the “full DNN”. We approach this in a two-pronged way. We summarize these two steps first and then give the details for each step.

Step 1: We analyze the weights of the edges in the full DNN and come up with the importance score of each input feature, which approximates how much the feature influences the final output of the DNN model. We then rank the features by their importance scores. We will refer to this rank-ordered list as “OL” (for, “ordered list”).

Step 2: From the OL, we take the top-*k* features and create a DNN with *k* input features and keeping the rest of the DNN architecture the same as the full DNN. We reduce the value *k* in steps of 20 and observe the drop-off in the validation rate. We find that the curve has a knee at a value of *k*, which implies that the accuracy degrades significantly if we use less than *k* features. We then use the top-k features for our experiments for the classifier which we call “reduced DNN”.

### Details of Step 1

The weights in a trained DNN contain information regarding the histone modification feature inputs and enhancers. In order to extract this information, we took a previous feature selection method [38] that determines feature importance from shallow Neural Network architecture connection weights, then expanded it for deep architectures and applied it to our initial-480 feature DNN. The equation used is given below, where *i* is a neuron whose importance score we are calculating, and *N*_*i*_ is the set of neurons in the next layer (closer to output) that *i* feeds into. The importance score of neuron *i*, denoted *S*_*i*_, is computed as

$$ {S}_i={\displaystyle \sum_{j\in {N}_i}\left|{w}_{ij}\right|{S}_j} $$

In this formulation, the neuron *j* is in the next layer to neuron *i* (i.e., closer to the output) and the weight of the edge that connects neuron *i* to neuron *j* is *w*_*ij*_. We start with the output neuron (a single neuron in our case) and set the importance of that neuron to 1. We then propagate the importance back to the previous layer’s neurons by using the weights of the edges connecting to the output neuron. The reader may notice that this is similar to the back propagation method, which is used to train DNN in the first place. Finally, we reach the input layer where each feature feeds into one neuron and the importance of the input neuron gives the importance of that feature.

### Details of step 2

Through step 1, we get an importance score for each input feature. We rank the features according to their importance scores in an ordered list OL (lowest importance features are at the end of the list). Then we start reducing the total number of features used by DNN by removing features at the end of OL. We train and evaluate DNN using the reduced feature set and compare its accuracy to that of the full DNN. In order to save time, we do this in steps of 20 features. We find that the accuracy is high and comparable to that of the full DNN till we get down to a certain number of features depending on the cell type.

Note that we make a simplification here with respect to training the reduced DNNs. We reuse the architecture of the full DNN in our reduced DNNs for all the internal layers (600-500-400 neurons) and do not optimize the architecture for each reduced set of features. The exact approach would have taken far too long since DNN training to come up with an optimized architecture is by far the most time consuming step. Empirically, we find that this simplification does not significantly affect the accuracy of the model.

## Results and discussion

A. Can a simple deterministic rule-based classifier work?

We asked ourselves the question: can a simpler deterministic, rule-based classifier tell apart the different forms of enhancers and between the enhancers and the non-enhancers? The classifier will use the same set of features that our EP-DNN uses, namely, the normalized expression values (RPKM) of the histone modifications. The intuitive way to structure such a classifier would be to consider the mean and the standard deviations of these features. These could be considered at the peak locations of these functional elements or also consider the bins around these adjoining regions. We experimented with both these approaches.

We show the mean and variance values for the 4 most discriminative histone modifications on H1 in Fig. 2 (these are for the peak locations of the respective functional elements). The main insight is that there is significant overlap in the expression values at the different functional sites. Thus, a simple classifier based on these features will be highly inaccurate in discriminating among the different classes. Even for the top histone modifications, the overlap between any positive type and unknown is significant, at 41.48 % or more. Taking the average of values of nearby locations does not help much, with the minimum overlap reducing only slightly to 39.69 %. While TSS has low overlap with other types for some histone modifications, TSS sites make up less than 0.05 % of all negative samples and are therefore not significant in the performance of the classifier.

These observations motivated us to look for more expressive classifiers and we settled on DNNs.

B. Validation rate of EP-DNN for 4 different cell types

**Fig 5:**
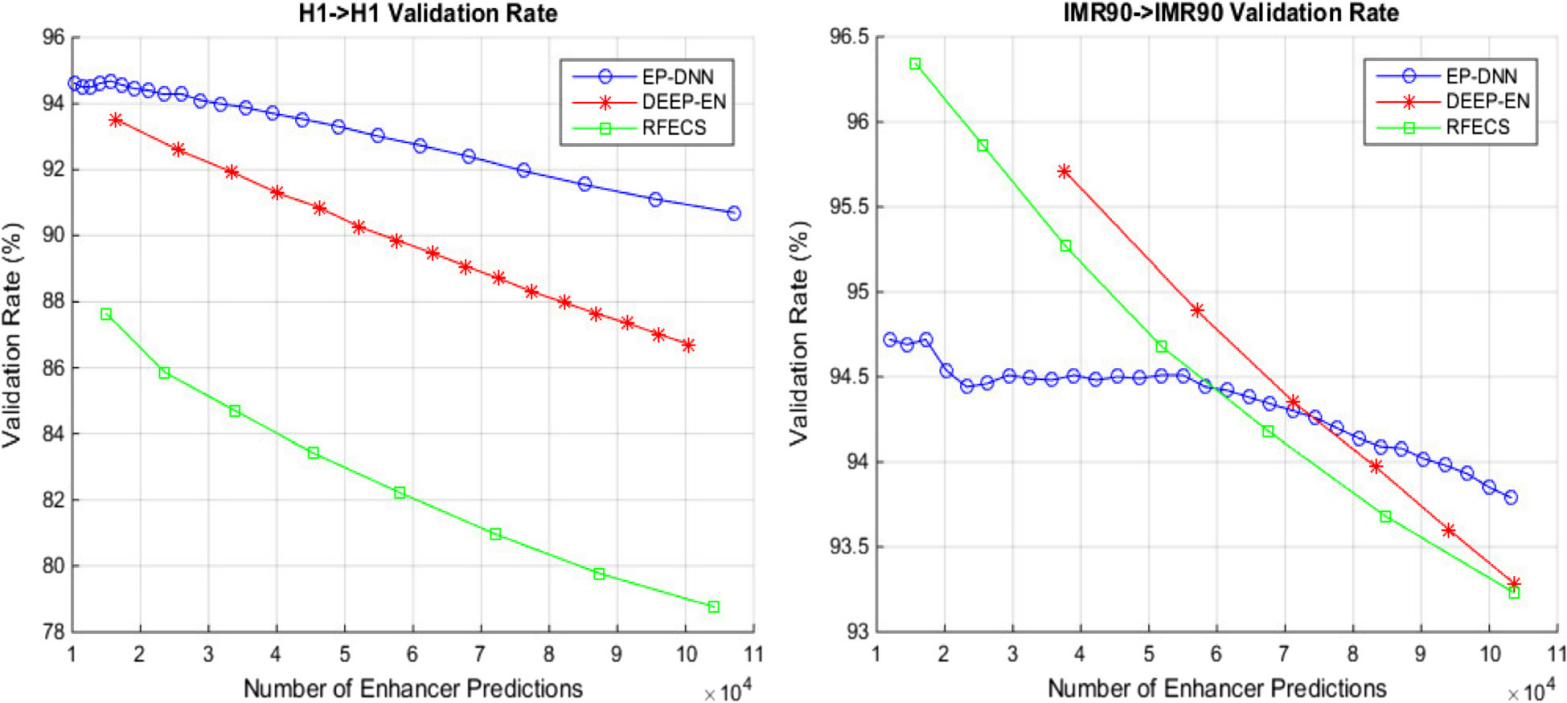
Comparison of EP-DNN against two state-of-the-art computational approaches for prediction of enhancer locations with respect to validation rate

Figure 5 shows the variation of validation rates for the three protocols—our protocol EP-DNN and the two recent protocols, DEEP-EN and RFECS—for the two cell types, H1 and IMR90. These are all same-cell predictions, i.e., we train on the same cell type as the one that we are trying to make predictions for. The DNN emits a numeric output, whose range may vary depending on what activation function is used, e.g., a sigmoid activation function results in the range [0, 1]. By varying the threshold parameter for the output from the DNN, we are able to get a varying number of enhancer predictions. Table 3 summarizes the result, for a fixed number of enhancer predictions, at approximately 100,000 enhancer predictions. The first and most important observation is that EP-DNN performs better for validation rates across the entire range of number of enhancers being predicted. (An exception is for IMR90-IMR90 validation, where EP-DNN performs better for high number of enhancer predictions, which we explain later.) Also note that the slope of the curve for EP-DNN is lower than for DEEP-EN and RFECS, implying that even when the protocol makes a large number of enhancer predictions, EP-DNN is more accurate. The only exception to the better performance of EP-DNN happens for IMR90 same-cell prediction, for high threshold values (*i.e.*, low number of predictions) where DEEP-EN and RFECS outperform EP-DNN. This likely happens because DEEP-EN and RFECS do a certain amount of overfitting to training data (DEEP-EN more so than RFECS) and such overfitting shows a (slightly) better prediction at high threshold values. This use case with high threshold values is arguably of use to experimentalists who are particular about high confidence predictions of enhancers for IMR90.

**Table 3:** Validation rates for the three protocols—our protocol EP-DNN and the two recent algorithms that define the state-of-the-art, DEEP-EN and RFECS, where we keep the number of enhancer predictions approximately constant, at 100,000

H1 Prediction	# of Predictions	Validation rate (%)
*EP-DNN*	104,994	90.76
*DEEP*	105,030	86.43
*RFECS*	104,155	78.76
IMR90 Prediction	# of Predictions	Validation Rate (%)
*EP-DNN*	103,196	93.79
*DEEP*	103,751	93.28
*RFECS*	103,624	93.23

Thus, this indicates that our EP-DNN model is more powerful as a classifier for datasets where the positive and negative examples may be more “inter-mixed”, and thus, harder to classify. This underlines a fundamental motivation for our use of DNN—the increased power of the model, at the expense of a greater effort in tuning the algorithm. Further, given that the H1 cell type is an embryonic cell type that is formative in character, it stands to reason that the differences between the signatures of enhancers and non-enhancers may be harder to resolve in it. We can contrast this to the adult cell type (lung fibroblasts) used in our study, IMR90, where these differences while easier to resolve by a classifier, presents a learning field that is devoid of the richness and subtleties of enhancer signatures presented by the embryonic cell type. The conclusion from the above scenario can be summed up as follows: first, EP-DNN is a better learning model; second, the H1 cell type (and possibly by extrapolation any other embryonic cell type) presents a harder learning task.

**Fig. 6:**
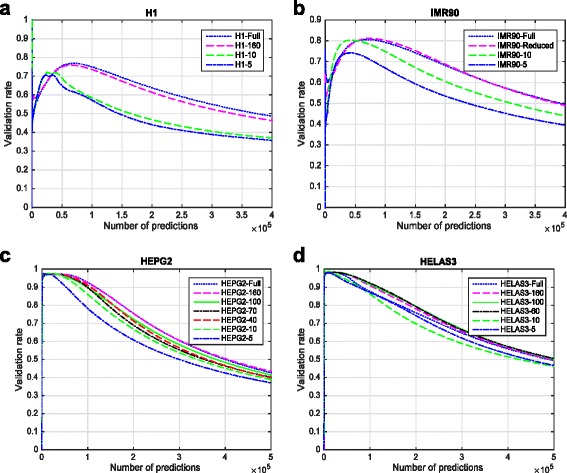
**a** and **b** Show the evaluation of EP-DNN with H1 and IMR90 for the full model and for a range of top-*k* values. **c** and **d** Show the evaluation of EP-DNN with two other cell types, HeLa and HepG2

Figure 6a and b shows the evaluation of EP-DNN with H1 and IMR90 for the full model and for a range of top-*k* values. Figure 6c and d shows the evaluation of EP-DNN with two other cell types, HeLa and HepG2. Here, we do not have results from RFECS and DEEP for a comparative comparison. In terms of relative performance of EP-DNN in these cell lines compared to H1, we see that the performance is better. This again speaks to the characteristics of the embryonic cell H1 where there is less differentiation among the different functional elements, making it harder for a model to differentiate between them.

### Validation rate summary table

We benchmark the validation rates for our technique along with two other state-of-the-art computational techniques (RFECS and DEEP-EN) for predicting enhancers, in Table 3. We evaluate the prediction of enhancers within a given cell line, individually for human embryonic stem cell line (H1) and a human lung cell line (IMR90). We keep the number of predictions by each technique to be close (approximately 100,000), for purposes of comparison. We find that DNN performs better in terms of validation rate. The advantage is more pronounced for prediction for the H1 cell type, where it is observed that enhancer prediction is a more difficult task than for IMR90. The improvement with DNN can be attributed to the use of the powerful DNN modeling technique, including multiple hidden layers and a large number of neurons at each layer, extensive feature selection, and optimization of the architecture and the parameters of the DNN.

### Validation rate detailed investigation

Upon detailed investigation into the factors that contribute to the validation rates, we find that the DHS that are distal from the TSS *and* the ones that are not p300, CBP, or TFBS (called *DHS-e*, “*e*” for enhancers), are the most numerous enhancers and provides the single largest contribution toward the validation rate. For IMR90, TFBS and CBP regions are not present in its dataset. The p300s and CBPs are more numerous in the data than the proportion in which they appear in our predictions. This can be explained by two factors. First, EP-DNN creates a model that generalizes well and does not overfit to the training data, which is all p300 for positive training examples, and consequently has a lower performance in predicting p300 sites. Second, the enrichment curves for p300s and CBPs are narrower, and thus, the signature may be weak toward the edge of the 2.5 kbp boundary from the enhancer peak location. The greatest contribution to the validation rate comes, as before, from the DHS regions that are *not* p300 binding sites but are enhancers. Note that we find that DNN is more prone to error in classifying some TSS sites as enhancers, more so than DEEP-EN and RFECS. However, the difference in TSS mis-prediction is not too significant between DNN and the others and TSSs are only a small fraction of the negative samples. Thus, in aggregate, the validation rates of EP-DNN are higher for the entire set of 4 cell lines.

C. Determining the most discriminative features

**Fig. 7:**
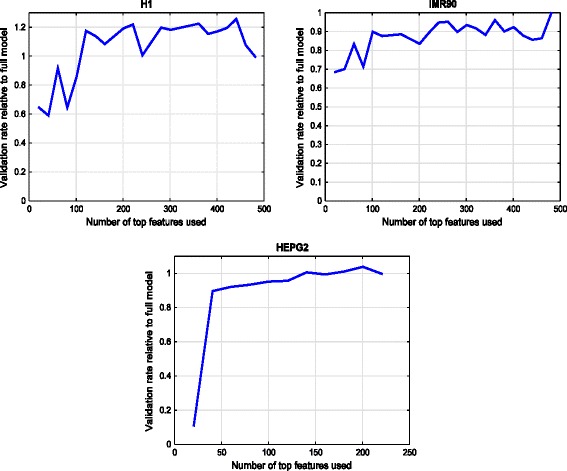
Feature importance score and validation rate when only a subset of features is used, for cell types H1, IMR90, and HepG2

We trained an initial DNN using all 480 features from the 24 histone modifications, then calculated the importance score for each feature from the learned weights of the DNN according to Equation 1. The features were then sorted by their respective importance scores and different subsets of features were used to train DNNs, starting with the top 10 features, top 20 features, and so on, ending at the full set of features. The architecture is fixed, with 400 neurons in the first layer, 300 in the second, and 200 in the third. Each DNN was tested against 5 random subsamples of 20 K samples each, chosen from among *all* the chromosomes. Figure 7 shows average subsample validation rate of each DNN, for H1, IMR90, and HEPG2 cell line, as we vary the number of features that the DNN takes as input.

We can see that validation rate increases sharply as important features are added. However, the rate increases more gradually after the top-150 features are added, for H1 and IMR90, and after the top-40 features are added for HEPG2. We omitted the HeLa plot because it looks similar to the HepG2 plot with the knee at the same point as well. This is interesting considering that the total number of features in HepG2 and HeLa S3 are different (220 versus 180), though they are close compared to the feature set for H1 and IMR90.. These results for the 4 cell lines confirm the validity of our weight analysis method and that it does indeed find the most important features for DNN and allows us to use a reduced subset of features for the final system. However, how big the reduced set should be is cell-type dependent.

Figure 6 shows the comparison between the full 480-feature DNN and the DNN with the selected top-*k* features for each of the 4 cell lines. For a given cell line, we plot the curves for different values of *k*. To generate this figure, we vary the threshold that is used as a comparison point for DNN’s raw output. Thus, as the threshold is raised, fewer number of enhancer predictions are made. An approximate determination of the realistic range for predicting prominent enhancer activity is when the validation rate is above 0.5; beyond that the predictions are too uncertain due to marginal enhancers or sites exhibiting weak enhancer signatures being predicted. Within this operational range, the validation performance with the reduced 160 features is no more than 5 % worse than with the full feature set. For much of the operational region, the difference is 2 % or less. Thus, we see that the reduction in the feature space, which reduces the cost of biological experiments to collect the data and the size of input data that a DNN has to be trained and tested with, does not hurt the enhancer prediction performance significantly. The interpretability of the DNN comes as another benefit of our process of reducing the feature set based on the importance scores of the features as calculated by our method.

We also see that if we reduce the number of features considered to be below that of the knee of the curve in Fig. 3 for any cell line, then the validation rate performance does fall significantly. For example, for H1, if we use only the top-10 features, out of the total of 480, then the validation rate drops by almost 20 % compared to the full model (at number of enhancer predictions = 1 × 10^5^). Similarly, for HepG2, when using only the top-5 features out of the total of 220 features, the validation rate suffers by 17.8 %. We can conclude that our sweep over the value *k* for the top-*k* features gives us a principled way of choosing how to subset the total feature set for training the DNN. This top-*k* applies to more data from the same cell line as well as in some cases across cell lines. For example, the knee of the curve is seen at approximately 160 for both H1 and IMR90. It remains a subject of future investigation to find out when the generalization of the top-*k* can be done across cell lines and for which specific cell lines.

D. Importance of the individual histone modifications for classification

**Fig. 8:**
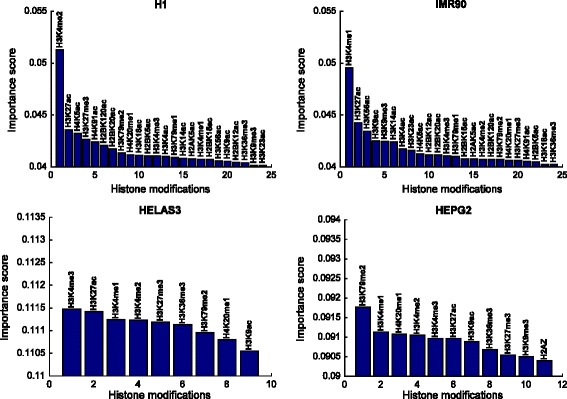
Importance scores of each histone modification, calculated as the sum of its 20 features. The important histone modifications are different for the four cell types, albeit with some overlap

**Fig. 9:**
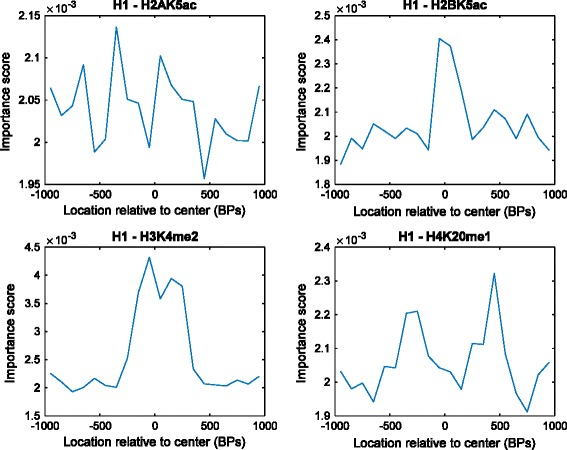
Importance scores for each feature of different histone modifications. The four specific histone modifications shown here represent the different patterns found through our analysis

Next, we find the importance of each histone modification by summing up the importance scores of its 20 features. The results are shown in Fig. 8. From this result, we take the top four histone modifications for each cell type and create a Venn diagram showing which histone modifications overlap with which cell types in Fig. 9. Due to the highly non-linear nature of the DNN, the absolute values of the calculated importance scores do not show the absolute magnitude of importance. However, the values do indicate the importance of a single histone modification with respect to the task of predicting enhancer. By comparing the importance values, we can rank the histone modifications.

The most important histone modifications according to our analysis confirm previous reports of H3K4me1 [31, 39], H3K27ac [40, 41], and H3K4me2 [16], being the most important ones, in various combinations, overall in global enhancer prediction. However, comparing the histone modification importance within each cell type reveals cell-type specific differences. While H3K4me2 and H3K27ac are the most important histone modifications for H1, for IMR90, H3K4me1 and H3K27ac are the most important. For HeLa S3, we see H3K4me3, also a known good predictor of enhancers, is the most important while H3K79me2 is the most important for HepG2. Although the well-known histone modifications (H3K4me1, H3K4me2, H3K27ac, and to some extent H3K4me3) place in the top important ranks, we can also see histone modifications are different for the cell types (with some overlap) in finer granularity. This information can help computational scientists when building models to make predictions on specific cell types. Further, it can also help life-science researchers optimize their experiments and collect the features for the most important histone modifications, for the cell type that they are focusing on.

E. Importance of the histone modification features for classification

Figure 9 shows importance scores of features within each histone modification. We selected four histone modifications to show the four distinct feature-importance patterns that we observe in the data. We omit the results from the other cell types since they have the same patterns as the H1 results presented here. This reveals that the most important features within a histone modification are not always centered at the enhancer site location, and consequently, it is detrimental to use fixed window sizes around the enhancer location, as all prior computational approaches have done. Window sizes that are too small can lead to important features being excluded, while large window sizes will include noise in the data that can be detrimental to prediction accuracy. Furthermore, certain “unimportant” histone modifications do contain relatively important features. This is why omitting histone modifications, altogether, even though they were reported to be unimportant can hurt the classifier’s performance. Thus, analysis at this finer granularity of features, rather than the coarser granularity of histone modifications used in prior approaches, is needed.

Sorting by the feature importance allows us to select only the most important and necessary features for prediction, instead of a fixed window size that has been used with previous methods. This allows us to reduce the number of input features necessary without a significant loss in its actual performance.

F. Visualization of the feature space

**Fig. 10:**
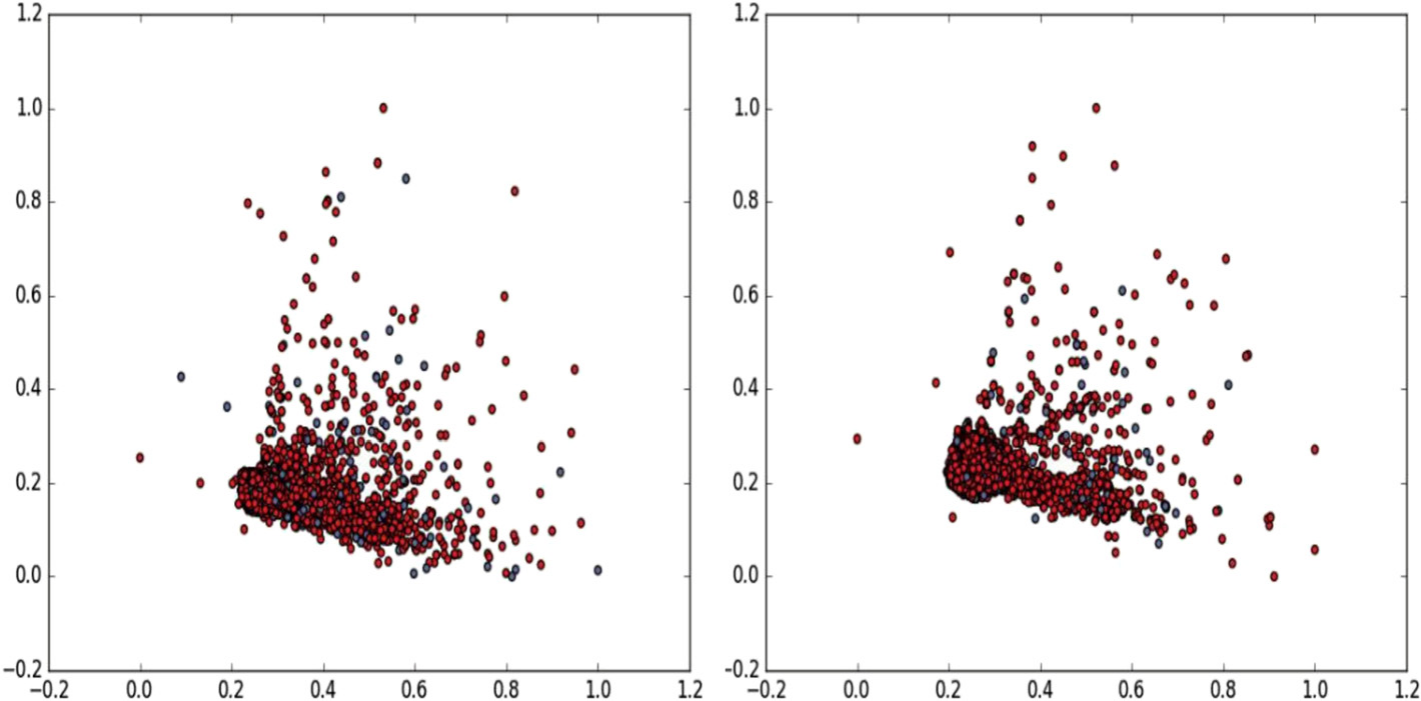
t-SNE visualization of the H1 cell line for all 480 features (left) and for the top 160 features, ranked according to their importance, i.e., discriminative ability (right). In both cases, the close clustering of the positive (blue circles) and the negative (red circles) samples show that it is a difficult task to separate them. Our DNN-based architecture, through its multi-layered structure and complex tuning procedure, is able to distinguish among the different kinds of positive elements (enhancers) and the different kinds of negative elements (non-enhancers)

To visualize the characteristics of feature space, we compute t-SNE transformation [45] with PCA initialization. First we compute PCA to create the 50 most important features and then we map them to a 2-dimensional space using the visualization method called t-SNE, a variation of Stochastic Neighbor Embedding [46]. This method tries to maintain the same distance between two points in the 2-d space as in the original (50 dimension in this case) space. This has been found to be an effective way to visualize high-dimensional data since it represents each data object by a two-dimensional point in such a way that similar objects are represented by nearby points, and that dissimilar objects are represented by distant points. The resulting two-dimensional map of the data that reveals the underlying structure of the objects, such as the presence of clusters. Figure 10 show the result of mapping feature spaces for all 480 features (left) and selected top 160 features (right) into 2-Dimensional t-SNE data, with red circles indicating the negative samples and blue circles the positive samples. Note that in the earlier experiment (Fig. 2) we were considering at the granularity of histone modifications; here we are considering the finer granularity of features, bins of histone modifications (recall that there are 20 of these for every histone modification).

From the t-SNE plots, we see that there is not a distinct separation between the positive and the negative samples. This further emphasizes that it is not easy for a simple rule-based classifier to separate the positive and the negative examples and motivates our use of a relatively sophisticated classifier like DNN. Our result, even after selecting the most important 160 features, does not show a clean separation between the positive and the negative examples [Fig. 10].

G. Execution time of the different models

In Table 4, we show the time to train and to predict using EP-DNN and EP-DNN-Reduced, for our current prototype implementation in Python, which underneath uses the toolkits Keras and Theano. These are done for the H1 cell line and the full model uses 480 features, while the reduced model uses 160 features. The training dataset has 40 K samples (positive: negative = 1:10) and the prediction is also done for 40 K samples. We also compare the runtime of our approach with DEEP and RFECS, both of which are implemented in Matlab. This comparison is not perfect because the implementations of these approaches take a smaller number of histone modifications than we do; DEEP-EN uses only 11 modifications and RFECS only 3 modifications. Since actual run times are highly dependent on several factors, such as the level of parallelization, hardware, platform, or implementation language, each method’s runtime was measured as the CPU clock time, under the same environment implemented in MATLAB2014rb (for DEEP and RFECS) and in Python (for EP-DNN-Full and EP-DNN-Reduced), with no parallelization. We acknowledge that some algorithms are more easily parallelizable than others and our method of using serial execution alone does not bring that aspect out. However, we followed this approach to take out the variability of different parallelization methods in order to compare the runtime results of the different protocols.

We see that a reduction from the full model to the reduced model of 1/3 of the features gives a slightly higher than proportional improvement to the training and the prediction times. RFECS has a much faster training time because it makes use of the highly efficient vectorized matrix computation of Matlab. In terms of the prediction time, which should be sped up as far as possible, EP-DNN-Reduced falls in between DEEP-EN (lower) and RFECS, with DEEP using 220 features (more than us) and RFECS using 60 features. However, DEEP performs poorly in terms of its training time and the time becomes infeasible for larger datasets.

## Conclusion

Enhancers are short DNA sequences that modulate gene expression patterns. Recent studies have shown that enhancer elements could be enriched for certain histone modification combinatorial codes, leading to interest in developing computational models to predict enhancer locations. However, prior attempts had suffered from either low accuracy of prediction or lack of interpretability of the results about which histone modifications are biologically significant. In this paper, we developed a DNN-based method, called EP-DNN, which addressed both of these issues. We find validation rates of above 90 % for the operational region of enhancer prediction for all four cell lines that we studied. The hardest to predict cell line is a human embryonic cell line called H1, possibly because the different functional elements are not fully differentiated in it yet, but EP-DNN outperforms DEEP-EN and RFECS, the two most recent computational approaches. Then, we developed a method to analyze a trained DNN and determine which histone modifications are important, and within that, which features proximal or distal to the enhancer site, are important. We uncovered that the important histone modifications vary among cell types, with some commonalities among them. We can then reduce the heavy computational cost of training a DNN by selecting the top-k features to use. We find that for H1, selecting a subset of 1/3 of the total set of features gives approximately the same validation rate as the full model, while reducing the computational time for training by a little more than 3X. Our results have implications for computational scientists who can now do feature selection for their classification task and for biologists who can now experimentally collect data only for the relevant histone modifications. In ongoing work, we are experimenting with parallelizing our computational approach and investigating further cell types to uncover possible groupings among cell types with respect to their enhancer characteristics.
